# Global Oral Health: Defining the Research Agenda in the Public Interest

**DOI:** 10.1177/00220345251372941

**Published:** 2025-10-18

**Authors:** J.E. Gallagher, C. Guarnizo-Herreño, D. Kavanagh, Y. Makino, M. Mathur, P. Mossey, B. Varenne, B. O’Connell

**Affiliations:** 1 Dental Public Health, King’s College London, London, UK; 2 Departamento de Salud Colectiva, Universidad Nacional de Colombia, Bogotá, Colombia; 3 Department of Health, Dublin, Ireland; 4 Noncommunicable Diseases Management Team, WHO Regional Office for Africa, Brazzaville, Congo; 5 Dental Public Health and Primary Care, Queen Mary University of London, London, UK; 6 Dundee University Dental School, Dundee, UK; 7 Department of Noncommunicable Diseases, Rehabilitation and Disability, World Health Organization, Geneva, Switzerland; 8 Faculty of Health Sciences, Trinity College Dublin, The University of Dublin, Dublin, Ireland

**Keywords:** dentistry, prevention and control, public health, population health, universal health insurance, world health organization

## Abstract

Research-informed action is central to the mission of the International Association for Dental, Oral, and Craniofacial Research (IADR). The IADR Presidential Symposium in Bogotá, Colombia, in June 2023 explored the regional research agenda to support the WHO Global Strategy and Action Plan on Oral Health 2023–2030. Facilitated by the Global Oral Health Inequalities Research Network and supported through the participation of World Health Organization representatives, this symposium involved researchers and policy makers across 4 regions. Each presented the unique strengths, challenges, and opportunities for their region, highlighting key topics for research and methodological approaches for future consideration. This article presents a series of contrasting regional perspectives to inform collaborative action among the global research community and strategic organizations. IADR’s national and regional associations can play a vital role in ensuring that by 2030, at least 50% of countries will have a national oral health research agenda focused on public health and population-based interventions, supported by the breadth of dental, oral, and craniofacial research to improve population health and facilitate universal health coverage.

**Report from the IADR Presidential Symposium, General Session, Bogotá, Colombia, June 2023**

## Introduction

The 2021 World Health Assembly resolution (WHA74.5; World Health Organization [WHO] 2021) reaffirmed that oral health should be part of the noncommunicable disease and universal health coverage (UHC) agendas. The resultant Global Strategy and Action Plan for Oral Health emphasize the importance of research to support the overarching targets of achieving UHC and a reduced disease burden for oral health (WHO 2024).

Strategic Objective 6 on research is pivotal, supporting all other strategic objectives:

SO6. To create and continuously update context- and needs-specific research that is focused on the public health aspects of oral health. (WHO 2024)

The global health research community has a major responsibility to push the boundaries of knowledge, inform evidence-based action, and support the reform of oral health care systems, driving the development and evaluation of more effective oral health policies and strategies across differing contexts and populations (WHO 2024). Furthermore, the action plan sets the following target for “research in the public interest”:

By 2030, 50% of countries have a national oral health research agenda focused on public health and population-based interventions. (WHO 2024)

This important agenda has implications for member states, international partners (which include the International Association for Dental, Oral, and Craniofacial Research [IADR]), civil society organizations, and the private sector. Despite the high prevalence and burden of oral diseases on the world’s population (WHO 2022), oral health does not receive sufficient priority in general health strategies and public health programs. Multiple reports, including those of the Lancet Commission on Oral Health (Lancet Commission 2023), the World Dental Federation (FDI) Vision 2030 (Glick et al. 2021), and the National Institute of Dental and Craniofacial Research (2021), have therefore been emphasizing that “now” is the time for action. Four regional perspectives presented at the IADR Presidential Symposium in Bogotá in 2023 are outlined here.

## African Region: Sub-Saharan Africa

Oral disease is prevalent across the WHO African Region (AFR), comprising 47 member states. AFR has the youngest and most rapidly growing population globally with 79% of people aged <30 y (United Nations 2022b); some 42% are estimated to have major oral diseases (WHO 2025). Furthermore, AFR bears the highest burden of “noma,” one of the most neglected tropical diseases globally (WHO AFR 2023). Despite having the largest increase in oral disease of any WHO region over the past 30 y, AFR has suffered from underinvestment in oral health. Half of its countries do not have oral health policies; >70% of countries spend less than US $1 per person per year on treatment costs for oral health care (WHO 2023); and there is a chronic workforce shortage in oral health (Gallagher et al. 2024).

### Priorities for Oral Health Research in the AFR

The Table outlines the lack of research supporting the creation, dissemination, implementation, monitoring, and evaluation of evidence-informed oral health policies, noting areas for action to accelerate the implementation of regional and global strategies on oral health. The latter focuses on population health and health services, including the health workforce, emphasizing the need to draw on models of workforce capacity building in collaboration (Bagg 2024; Urquhart et al. 2024).

**Table.** Research in Support of Oral Health by Region: Strengths, Challenges, Opportunities, Methodology, and Topics.

Existing Strengths	Presenting Challenges	Opportunities to Embrace	Methodological Research Approaches Advocated	Topics: Key Issues to Address
**African Region** 47 countries (45% urban at continent level) ^a,b^				
Existence of regional and global strategies on oral health highlighting the importance of research Regional project on research prioritization on oral health	Underinvestment in oral health Much research driven by external partners Research does not address national health priorities Unclear results related to impact on priority health problems Weak sharing and utilization of the research results Significant workforce challenges, including training and retraining researchers	Increase the number of oral health research institutions Progress for general research for health situation (e.g., the proportion of countries with a research policy or a budget line for research for health increased from 51% to 62% and from 51% to 67% between 2014 and 2022, respectively ^c^) Integration of oral health into integrated surveillance system such as WHO STEPS (e.g., 10 countries conducted the oral health module of the WHO STEPS after 2016 ^d^ and used the results to develop their national oral health policies and strategies)	Research studies informing the effect of interventions to create locally tailored solutions to oral health issues and the assessment of the implementation and evaluation of those solutions Evidence synthesis, including systematic reviews and policy briefs as essential bricks, to support the creation of national and regional oral health policies ^e^ Collaborative research involving regional and national actors Implementation research	Innovative models of oral health care involving universal health coverage and primary health care Building regional/local research oral health workforce capacity Determinants of oral health in the African Region Addressing national health priorities in collaboration Sharing and utilizing research evidence Implementing evidence-informed solutions and evaluation
**Latin American and Caribbean Region** 33 countries (82% urban) ^a,b^				
Participatory action research approaches	Stubbornly high levels of exclusion and socioeconomic inequality, with Latin American and Caribbean countries exhibiting higher-income inequality than those in other regions at similar development levels Conflicts of interest and profit motives behind research agendas Funding opportunities for research Public policies continue to be largely based on individual risk factors with a strong emphasis on behavioral change Underfunded and overburdened health care systems	Given the recent changes in the general approach to social policies that are taking place in Chile, Colombia, Brazil, among others, it would be very relevant to study their potential impact on population oral health and inequalities	Community involvement Methodological approaches involving participatory action approach Codesign/coproduction of interventions	Models of delivering quality, equitable dental care Novel technologies, including those derived from codesign/coproduction of interventions Understanding the mechanisms that produce and reproduce oral health inequalities Understanding commercial determinants of oral health and their impacts in the region Monitoring the use of fluorides at the community level (water or salt fluoridation) Integration of oral health in general primary care initiatives
**South East Asia Region** 11 countries (53% urban) ^a,b^				
Thriving hub for IT-driven health care innovation designing, evaluating, and scaling up health care models, leading to cost-effective, impactful solutions for various health issues Well-defined primary health care systems Large skilled workforce	Large and diverse population Growing burden of NCDs High prevalence of head and neck cancer Persistent challenges in maternal and child health, mixed health systems, low public financing, high rates of infectious diseases and child undernutrition Substantial out-of-pocket health expenditure by population	Hub for IT-driven health care Proven effective community-involving health promotion and care models Government buy-in for SDGs and universal health coverage as a political priority Successful implementation of many NCD programs, especially tobacco control Global leadership in encouraging South-South knowledge exchange and cooperation	Codesigning of oral health promotion interventions, especially involving the most deprived populations Development of surveillance tools for capturing the burden of oral diseases and systems-based indicators for oral health Advanced analytic methods to synthesize large population-based datasets for oral diseases Development of population cohorts	Codesigning interventions for improving oral health of the most deprived communities Analyzing big data for more robust evidence on oral health Integrating oral health in universal health coverage and SDG strategies Addressing the commercial determinants of oral health Health systems strengthening for delivering appropriate oral health services Frugal innovations for oral health care
**European Region** 53 countries (75% urban) ^a,b^				
Availability of data, including national and regional general and oral health surveys and census Oral health accepted by European Region as an integral part of NCDs Substantial workforce capacity and skill mix levels available Dental caries is reducing in children Strong tobacco public health policies and reductionExample of comprehensive mandatory alcohol labelling in one MS Established research community with focused funding provided by EU (EU4Health and HORIZON), including a public health focus	Aging population with less research available vs younger cohorts Treatment-based health care systems that require high resources that are not sustainable for many countries Highest alcohol consumption of all other WHO regions Rising use of vapes (nicotine) with unclear oral health impacts High tobacco use in younger femalesBurden of disease increasing with population expansion: dental caries, tooth loss, oral cancer/head and neck cancer Oral health workforce: uneven distribution, changing career priorities, retention	Managing an aging population Harnessing the knowledge of an older workforce for mentoring and research Innovative models of care involving skill mix, including expansion of roles to broader NCD agenda supported by digital technologies Greater public involvement in research Reorientation to prevention supported by dental amalgam phaseout legislation in the EU	Longitudinal cohort research Innovative surveillance models Workforce modeling Integration of oral health with general health and other NCD-related research	Oral health in an aging population Impact of sugar policies and evaluating interventions to reduce volume of sugar intake Impact and use of vaping (e-cigarettes) Tobacco reduction in females requires greater focusAlcohol consumption, especially binge drinking Understanding and addressing inequalities within and between countries in oral health services Integration of NCD services (e.g., diabetics services and oral health care) Impact of prevention on use of antibiotics

Table provides an overview of population research strengths, challenges, opportunities, methodological approaches, and key issues across 4 regions.

EU, European Union; IT, information technology; MS, member state; NCD, noncommunicable disease; SDG, sustainable development goal; STEPS, STEPwise Approach to NCD Risk Factor Surveillance; WHO, World Health Organization.

a WHO regional offices (who.int).

b Statista (statista.com).

c WHO Regional Office for Africa (2023).

d WHO Regional Office for Africa (2025).

e Labarca et al. (2024).

While the AFR Oral Health Strategy has been addressing oral diseases as part of noncommunicable diseases for some time (WHO AFR 2016) and 11 of 47 countries have reported the establishment of partnerships to develop and implement research (WHO AFR 2022), many countries have reported a lack of evidence, research, monitoring, and evaluation systems. Therefore, building evidence to support decision making, inform action, and track progress in oral health activities will be fundamentally important.

## Latin American and Caribbean Region

The Latin American and Caribbean Region (LACR) encompasses a population of approximately 670 million people (United Nations 2022a). It faces numerous challenges, including persistent regional inequality that results from a complex combination of colonial legacy, economic systems, and dynamics of state-society interactions (Ystanes and Strønen 2017; Amorim et al. 2019; United Nations 2021). Health care systems are often underfunded and overburdened, corruption is relatively high, and population migration presents additional challenges (Sampaio et al. 2021). Commercial determinants of oral health in general and sugar consumption are particularly relevant for this region, which supplies about 40% of the global sugar output and has a strong historical relationship with its production and consumption (Organisation for Economic Co-operation and Development 2019). There has been very strong opposition by industry to regulations such as health taxes and front-of-package nutrition labeling. Despite these realities, some recent positive advances have been observed. For example, Brazil has seen an exponential increase in primary oral health care teams (about 30,000 in 20 y) in the context of the Unified National Health System, the Sistema Único de Saúde, which also encompasses a strong monitoring and evaluation system (Pucca et al. 2015). Additionally, Chile, México, and Colombia have successfully implemented regulations aimed at reducing consumption of unhealthy food and beverages (Taylor 2023; Gómez 2025).

### Priorities for Oral Health Research in the LACR

Oral diseases are prevalent with high levels of socioeconomic inequality (Guarnizo-Herreño et al. 2019; Sampaio et al. 2021; Soares et al. 2019). Research-informed action on tackling the determinants of oral health, especially political influences, is urgently needed. Future research should embrace wider methodological challenges (Table). The research agenda should also include monitoring the use of fluorides at the community level (water or salt fluoridation) and testing ways to advocate for improved access to basic amenities such as drinking water and sanitation. In addition, research should be aimed at understanding access barriers to dental care and poor infrastructure, which particularly affect those residing in rural areas and living in vulnerable socioeconomic conditions. Moreover, studies on models of delivering quality dental care are key for the region, as well as for evaluation of innovative approaches and novel technologies, including those derived from codesign/coproduction.

Some additional challenges and topics comprise the following: first, conflicts of interest and profit motives behind research agendas; second, how political decisions are made (e.g., why public policies continue to be largely based on individual risk factors with a strong emphasis on behavioral change); and third, given the recent changes in the general approach to social policies that are taking place in Chile, Colombia, Brazil, among others, it would be relevant to study their potential impact on population oral health and inequalities.

Oral diseases disproportionally affect excluded groups in societies (Celeste and Nadanovsky 2009; Guarnizo-Herreño et al. 2019; Soares et al. 2019), and such communities have developed their own meaning, perception, and actions for coping with the oral health issues determined by their living and working conditions (Banerji 2006). Listening to their voices is key, and understanding the mechanisms that produce and reproduce the observed oral health inequalities is essential to achieve meaningful community engagement and policy change. For example, the participatory action research framework challenges existing dominant power relations, colonial legacies, and how these perpetuate health/oral health inequalities (Fals Borda 2001; Rumsey et al. 2022). The LACR has strengths in using this type of approach, and using it to better understand and address inequalities in oral health would be a significant step forward.

## South-East Asia Region

The South-East Asia Region (SEAR) encompasses a population of approximately 2 billion people, accounting for roughly 25% of the world’s population (WHO SEAR 2024). This is a region of great importance for public health research (Table). The Action Plan for Oral Health in SEAR (2022–2030) outlined 2 critical targets: a 33.3% reduction in premature oral cancer–related mortality and a 25% reduction in untreated dental caries in permanent teeth by 2030 (WHO SEAR 2023).

SEAR has unanimously adopted the sustainable development goals (United Nations High Commissioner for Refugees 2015) in their policy discourse for 2030 (*The Lancet* 2023). A major roadblock that countries encounter is the paucity of disease-specific data, including oral diseases. Thus, key themes, such as data, workforce, UHC, integration, and frugal innovation, are foundational in health care and global development.

### Priorities for Oral Health Research in SEAR

The main research priorities are presented in the Table. UHC in SEAR is contingent upon the inclusion of oral health in a strengthened primary care model. Seeking answers to key research questions can advance oral health care—such as tailoring primary care reform models for oral health care; integrating insurance to cover primary and preventive oral health, including outpatient services in health care coverage; defining the essential package of oral health services; adopting technology; advocating for professional organizations; and actively engaging communities and the public in health care decisions.

SEAR is a thriving hub for information technology–driven health care innovation. The region has excelled in designing, evaluating, and scaling up health care models, leading to cost-effective, impactful solutions for various health issues, and this energy must now be used to address oral diseases.

Strengthening oral health research systems in SEAR requires a focused commitment to robust data collection, integration, and analysis. Accurate, comprehensive data are essential for shaping informed policies, planning equitable resource distribution, and effectively advocating oral health’s integration into UHC and noncommunicable disease frameworks and datasets. Given ongoing regional discussions around UHC, prioritizing oral health interventions that adopt an upstream approach—addressing social determinants of health, preventive measures, and community-based strategies—is crucial. This proactive model has potential to not only maximize health impact but also enhance sustainability and equity in oral health care delivery across the region.

## European Region

The WHO European Region (EUR) has a population of 923 million people across 53 countries (WHO EUR 2024). The demographic profile is changing rapidly such that by 2050 people aged ≥65 y will represent 25% of the population. Inequalities are evident with southern and eastern areas less affluent (Eurostat 2023a). Thus, educated professionals, including dentists, tend to migrate to northern Europe to earn more money.

Noncommunicable diseases are the main cause of premature mortality and cause 83% of premature deaths. Since 2010, tobacco use has declined, but its reduction is variable across member states, where its use is 2 to 3 times greater among women than in other regions. Research indicates that e-cigarettes are emerging as a new risk factor, and the region has the highest consumption of alcohol globally (WHO EUR 2022a). New oral cancer cases in EUR account for 18.5% of cases globally (WHO EUR 2023). Furthermore, it has the highest prevalence of tooth decay in permanent teeth at 33.6%, with 12.4% prevalence of tooth loss in people aged >65 y; on a positive note, since 1990 the prevalence of caries in children’s teeth has declined (WHO EUR 2023).

Vast inequalities in access to oral health services exist within and among countries. People with the greatest need are less likely to be able to afford to access care such that those in isolated, rural, and deprived areas are most affected (Eurostat 2023b). Patients bear significant out-of-pocket costs for dental care in Europe, and even in wealthy European countries, there are disparities in access to care (WHO EUR 2023).

Regional strengths and weaknesses are summarized in the Table. First, despite its large workforce (Gallagher et al. 2024), impending retirements of an aging workforce in parts of Europe present a major challenge (WHO EUR 2022b). Second, well-established European public and private dental systems are historically intervention focused and will take time to effectively reorient toward prevention. The development of dental team skill mix and task sharing with other primary care professionals will facilitate movement beyond traditional approaches to dental care and have the potential to increase workforce capacity.

The European WHO report outlines 4 key asks of member states: these include the need to focus on governance, promotion and prevention, oral and primary health care services, and workforce (WHO EUR 2022a).

## Call to Action

While oral health research is crucial for improving health care in every region, research requires sustained investment, collaboration, and innovative solutions as part of wider health strategies. Efficient cooperation between countries would be beneficial in relation to tackling well-known common risk factors and upstream determinants of noncommunicable diseases, particularly for oral health. Although not a comprehensive list of challenges and topics for oral health research, the material from the Bogotá symposium provides nuanced and useful insights for discussion among researchers, policy makers, activists, and other relevant stakeholders. Research forms a pivotal role in the adaptation and implementation of the Global Oral Health Action Plan (WHO 2024) to improve oral health and achieve universal access to essential care across all income settings. There is an urgent need for collaborative research-informed action on global issues, from phasing down the use of dental amalgam within environmentally sound oral health care systems to tackling antimicrobial resistance, embracing digital technologies, and developing public health programs and population-based initiatives initiatives for oral health (WHO 2024).

IADR’s national and regional associations will play a key part in ensuring that countries have a national oral health research agenda focused on relevant public health and population-based interventions. This should be supported by the breadth of research, from discovery through applied science, with the aim of better oral and general health and universal access to care. It should also embrace the spectrum of research, from individuals to communities to health systems, and support long-term sustainability.

As an academic community, IADR members can play a vital role in influencing policy makers locally, regionally, and globally. We should encourage coming generations of researchers to apply their skills and energy for the improvement of oral health on a global scale and highlight the best opportunities for research to support the WHO’s shared goals. A measure of our success will be the empowerment of researchers across all regions to address the critical questions of most relevance to their populations and collaborate on addressing key global topics in partnership.

## Author Contributions

J.E. Gallagher, contributed to conception and design, data acquisition and interpretation, drafted and critically revised the manuscript; C. Guarnizo-Herreño, D. Kavanagh, Y. Makino, M. Mathur, contributed to design, data acquisition, analysis, and interpretation, drafted and critically revised the manuscript; P. Mossey, B. Varenne, contributed to conception and design, data interpretation, critically revised manuscript; B. O’Connell, contributed to conception and design, data interpretation, drafted and critically revised manuscript. All authors gave their final approval and agreed to be accountable for all aspects of the work.
